# Biomechanical comparison of lag screw and non-spiral blade fixation of a novel femoral trochanteric nail in an osteoporotic bone model

**DOI:** 10.1038/s41598-022-04844-5

**Published:** 2022-01-17

**Authors:** Yoshifumi Fuse, Yukichi Zenke, Nobukazu Okimoto, Toru Yoshioka, Yoshiaki Yamanaka, Makoto Kawasaki, Hiroshi Terayama, Akinori Sakai

**Affiliations:** 1 Department of Orthopaedic Surgery, Saka Midorii Hospital, 6-28-1, Midorii, Asaminami-Ku, Hiroshima Shi, Hiroshima Ken 731-0103 Japan; 2 Department of Orthopaedic Surgery, University of Occupational and Environmental Health, 1-1, Iseigaoka, Yahatanishi-ku, Kitakyusyu-shi, Fukuoka, 807-8555 Japan; 3 Okimoto Clinic, 185-4, Yutakamachi Kubi, Kure Shi, Hiroshima Ken 734-0304 Japan; 4 Department of Orthopaedic Surgery, Shimura Hospital, 3-13, Funairimachi, Naka Ku, Hiroshima Shi, Hiroshima Ken 730-0841 Japan

**Keywords:** Trauma, Experimental models of disease, Geriatrics

## Abstract

There is no consensus regarding the advantages of the lag screw type over the blade type for treating femoral trochanteric fractures. We aimed to investigate whether non-spiral blade (Conventional-Blade, Fid-Blade) nails provide better biomechanical fixation than lag screws in a severe osteoporotic bone model. Different severities of osteoporotic cancellous bone were modelled using polyurethane foam blocks of three densities (0.24, 0.16, and 0.08 g/cm^3^). Three torsional tests were performed using each component for each density of the polyurethane block, and the maximum torque was recorded; subsequently, the energy required to achieve 30° rotation was calculated. Using a push-in test, the maximum force was recorded, and the energy required to achieve 4-mm displacement was calculated. For 0.08-g/cm^3^ density, the peak torques to achieve 30° rotation, energy required to achieve 30° rotation, peak force to achieve 4-mm displacement, and energy required to achieve 4-mm displacement were significantly greater for Conventional-Blade and Fid-Blade than those for Lag Screw. The fixation stability of the blade-type Magnum nail component is better than that of the lag screw type under any test condition. The blade-type nail component may have better fixation stability than the lag screw type in a severe osteoporotic bone model.

## Introduction

Hip fractures among the elderly are major fragility fractures, and early treatment and ambulation are necessary. It has been reported that the most serious social and economic concerns faced by Japan’s health care system is the rapid rise in the number of hip fractures in individuals aged ≥ 90 years^1^. Femoral trochanteric fractures are more closely associated with a low bone mass than are femoral neck fractures, and they occur more frequently in very old individuals^2^. In very old patients, early ambulation without weight-bearing is almost impossible. Therefore, stable fixation of hip fractures is crucial. Intramedullary nailing is the gold standard for treating unstable femoral trochanteric fractures, with good efficacy and outcomes^3,4^. However, the osteoporosis severity influences cutting out of the nail after femoral intramedullary nailing^5^; consequently, the number of cases of femoral trochanteric fractures, where stable fixation using conventional methods is difficult, may increase for several decades.

Surgeons can choose between two types of intramedullary nails, namely the lag screw and blade types, for femoral head fixation in femoral trochanteric fracture treatment. The blade type was developed to increase the holding capability of the implant in the femoral head and lower the cut-out rate^6^; however, there is no clear consensus regarding the advantages of the lag screw type over the blade type. The conventional helical blade is inserted using a rotational movement, and this rotational movement can cause rotational dislocation of the head, unlike the lag screw, which allows fine rotational adjustment. Conversely, the non-spiral blade may improve cancellous bone compaction and provide better holding capability, even in the osteoporotic femoral head, by driving the blade into the femoral head without pre-reaming and rotating. However, there is paucity of data on the biomechanical properties of the non-spiral blade in severe osteoporosis.

Accordingly, we aimed to investigate the differences between the lag screw and blade type in bone models with different densities, particularly to verify whether the fixation capability of the non-spiral blade type is better than that of the lag screw type in a severe osteoporotic bone model.

## Methods

**Figure 1:**
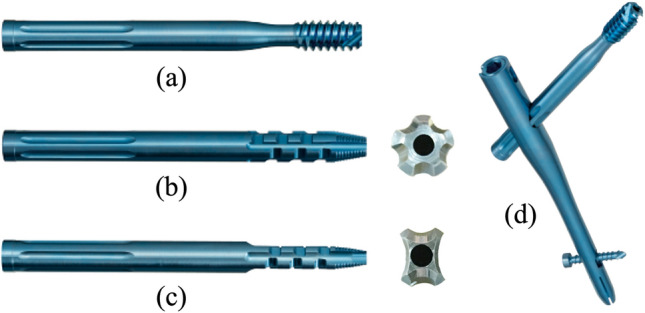
(**a**) Lag Screw component. (**b**) Conventional-Blade component, showing its cross-sectional shape. (**c**) Fid-Blade and its cross-sectional shape (two-face-cut design). Both blades are driven into the femoral head without rotation. (**d**) Magnum nail.

We used three components of the Magnum nail (Robert Reid Inc., Tokyo, Japan), which is a nail-type device for improving rotational fixation in severe osteoporosis; this nail can utilise both a lag screw (termed Lag Screw) and blade components in various sizes for femoral head fixation, and the surgeon can choose among the three components during the surgical procedure^7^. Two blade-style components, termed Blade (Conventional-Blade) and Fid Blade (Fid-Blade), exhibiting a non-spiral shape, are available (Fig. 1). Conventional-Blade has a convex and concave shape, with several longitudinal grooves that prevent it from backing out, while Fid-Blade has large face shapes on the lateral surfaces that enhance rotational resistance. Moreover, Fid-Blade has fewer longitudinal grooves; hence, the nail can be easily pulled out in younger patients.

Polyurethane foam blocks (Sawbones, Vashon Island, WA, USA) were used as a bone substitute material to simulate the osteoporotic cancellous bone in the femoral head^8^. Owing to its uniform structure with only ± 10% density variation^9^, foam blocks provide a consistent material with characteristics in the range of a human cancellous bone. These blocks are 40 mm in length and width and 50 mm deep. Blocks of three different densities were used as follows: 0.24 g/cm^3^ for normal bone mineral density, 0.16 g/cm^3^ for bone with mild osteoporosis, and 0.08 g/cm^3^ for bone with severe osteoporosis^8^. Three blocks of each density were prepared for each of the Lag screw, Conventional-Blade, and Fid-Blade type nails. To evaluate the fixation capability of the implants, torsional and push-in tests were performed.

**Figure 2:**
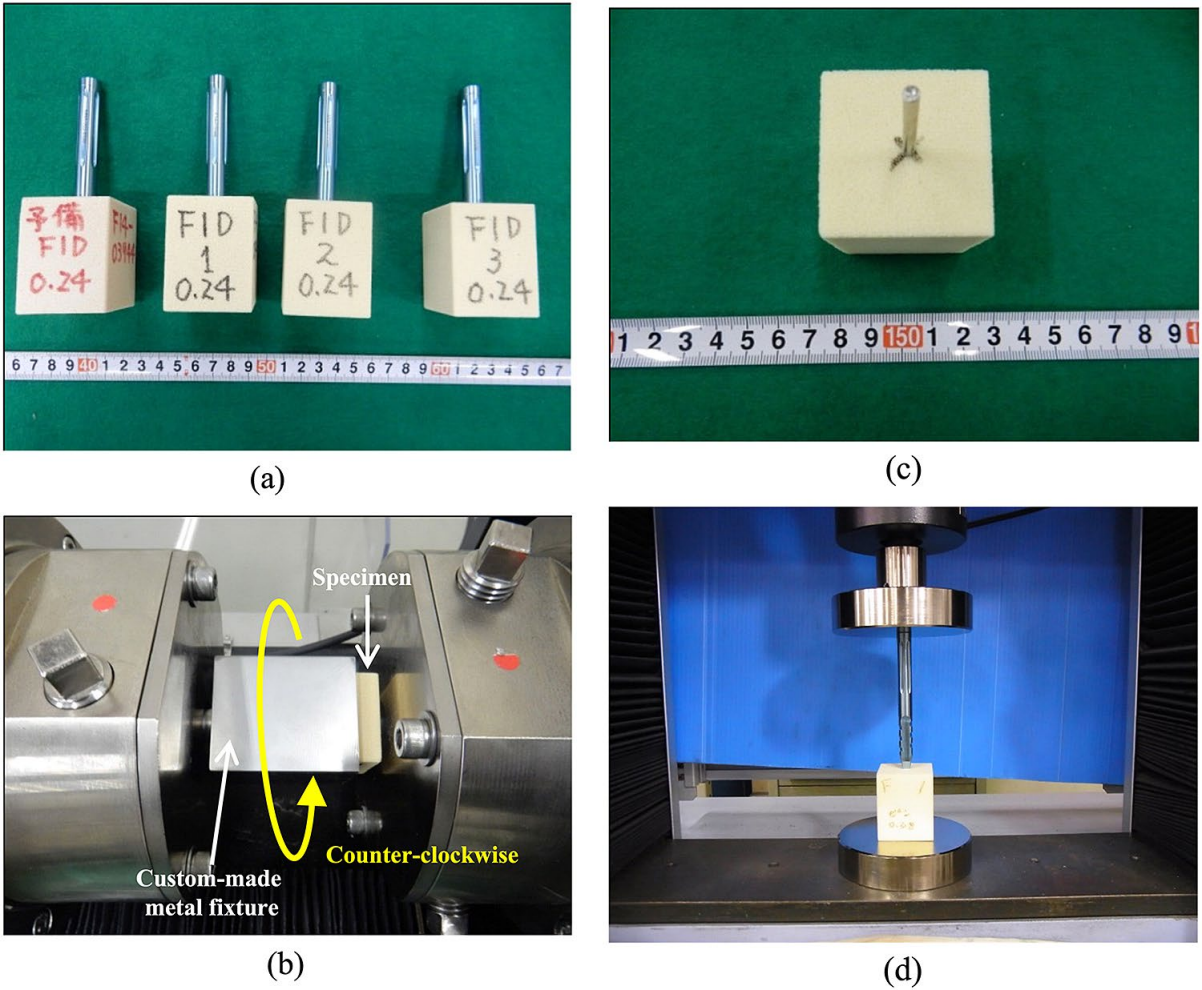
(**a**) Specimens of the three densities of the polyurethane block: 0.24, 0.16, and 0.08 g/cm^3^. (**b**) Shimadzu Torsional Testing machine. (**c**) Specimens inserted to a depth of 40 mm in advance. (**d**) Autograph Tensile Testing machine.

Figure 2a,b illustrate the specimens and test setup of the torsional test, respectively, used to measure the rotational resistance of each component. The torsional test was performed three times with each of the three components and each of the three densities of the polyurethane block. Each component was inserted to a depth of 40 mm (assuming a depth of 10 mm from the subchondral bone) in advance (Fig. 2a). The tests were performed on a Shimadzu Torsional Testing machine (Shimadzu Corporation, Kyoto, Japan; Fig. 2b) at 24 °C. First, the polyurethane block was inserted into the custom-made metal fixture and then the component was fixed. Subsequently, torque was applied to the custom-made fixture containing the polyurethane block. Finally, the polyurethane block and component were rotated counter-clockwise and clockwise, respectively, with a constant angular velocity of 1/60 s-1 while measuring the torque. The implant was only loaded with torque around the axis of the component. Individual torque–angle curves and the maximum torque as rotational resistance were recorded until 30° of component rotation for each test. The resistance of the torsional test was calculated as the energy required for each implant to achieve 30° rotation, which corresponded to a 10-mm linear displacement. The energy values were determined by calculating the area under the torque–angle (in radian) curves.

Figure 2c,d illustrate the specimens and test setup of the push-in test, respectively, to measure resistance during the insertion. The push-in test was conducted three times for each of the three components for each of the three densities of the polyurethane block. Tests were performed on an Autograph Tensile Testing machine (AG–X 5.0 kN, Shimadzu Corp, Kyoto, Japan; Fig. 2d) at 24 °C. All components were inserted with a constant angular velocity of 20 mm/min while the force was being measured. In this test, all components were inserted over a 3.2-mm guide pin to a depth of 40 mm in advance (Fig. 2a); then, individual force–displacement curves and the maximum force were recorded until the depth of 44 mm was reached for each test. The resistance encountered in the push-in test was calculated as the energy required for each implant to achieve a 4-mm displacement. The energy values were determined by calculating the area under the force–displacement curves.

Statistical analysis was performed by using a *t*-test in the statistical software JMP ver. 12.2.0 (SAS Institute Inc., Tokyo, Japan). *P*-values were calculated using one-way analysis of variance with Tukey’s HSD test, and *P*-values < 0.05 were considered significant.

### Ethical approval

Approval from the Institutional Review Board was not required because this study did not include any human participants or data.

## Results

**Figure 3:**
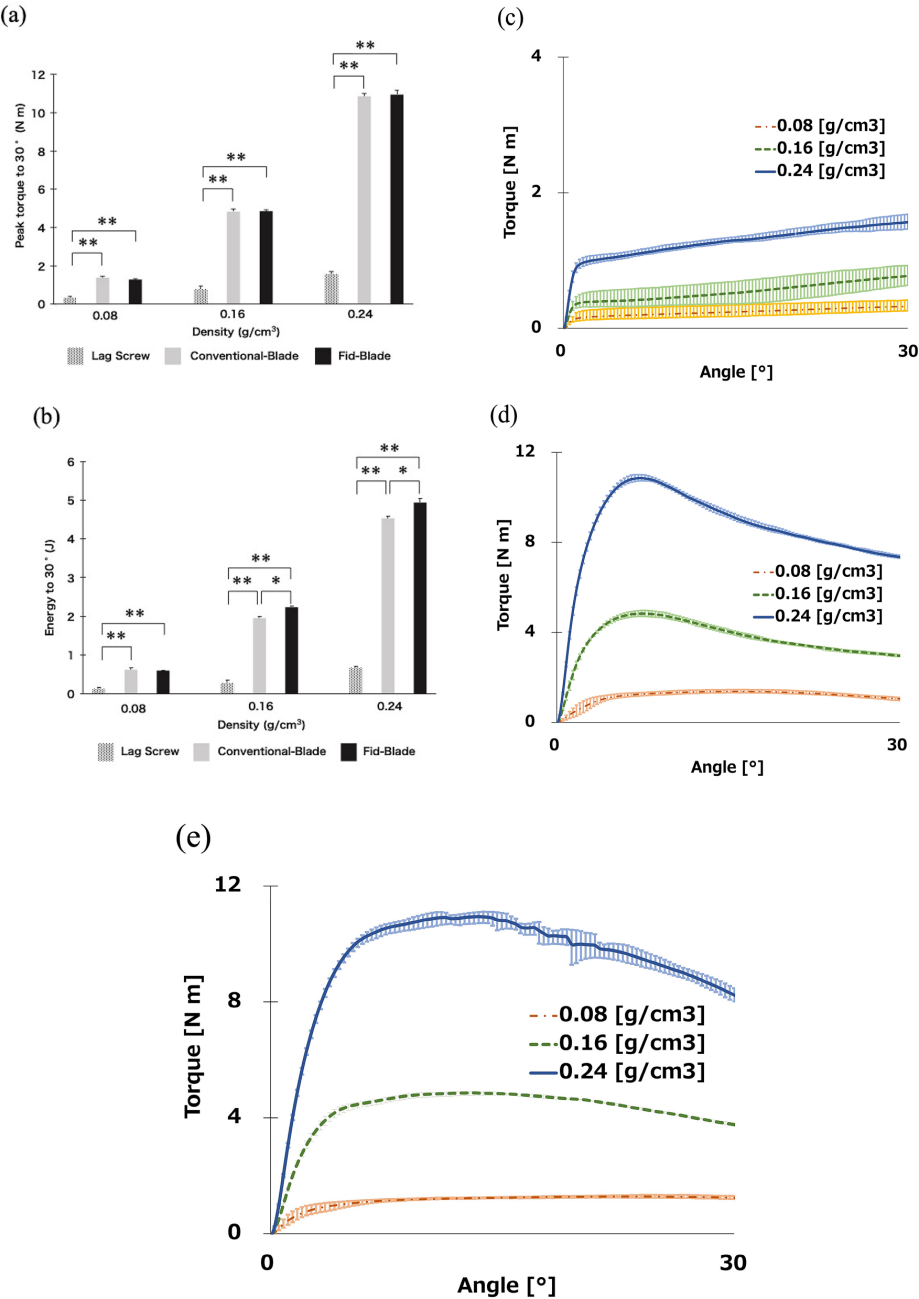
Torsion test. (**a**) Mean peak torque to achieve 30° rotation. (**b**) Mean energy to achieve 30° rotation. *P < 0.05. **P < 0.01. (**c**–**e**) Mean torque–angle curves (**c**) Lag Screw. (**d**) Conventional-Blade. (**e**) Fid-Blade.

The peak torques and energy to achieve 30° rotation in the torsion test are shown in Fig. 3a,b, respectively. The lower the density of the polyurethane block, the lower the peak torque and energy value. At a density of 0.08 g/cm^3^, the peak torques to achieve 30° rotation were significantly greater for Conventional-Blade (1.38 ± 0.07 Nm) and Fid-Blade (1.28 ± 0.04 Nm) than those for Lag Screw (0.33 ± 0.07 Nm; *P* < 0.01, *P* < 0.01), with no significant differences between Conventional-Blade and Fid-Blade. The energy required to achieve 30° rotation was also significantly greater for Conventional-Blade (0.62 ± 0.04 J) and Fid-Blade (0.60 ± 0.01 J) than that for Lag Screw (0.13 ± 0.04 J; *P* < 0.01, *P* < 0.01), with no significant difference between Conventional-Blade and Fid-Blade. During the torsion test, both Conventional-Blade and Fid-Blade showed significantly higher values than Lag Screw in all densities of the polyurethane block. The mean torque–angle curves of the torsional test are shown in Fig. 3c–e. Conventional-Blade and Fid-Blade achieved a higher peak torque value with minimal rotation than Lag Screw, and they maintained the high value even after rupture, whereas Lag Screw continued to rotate at low torque values.

**Figure 4:**
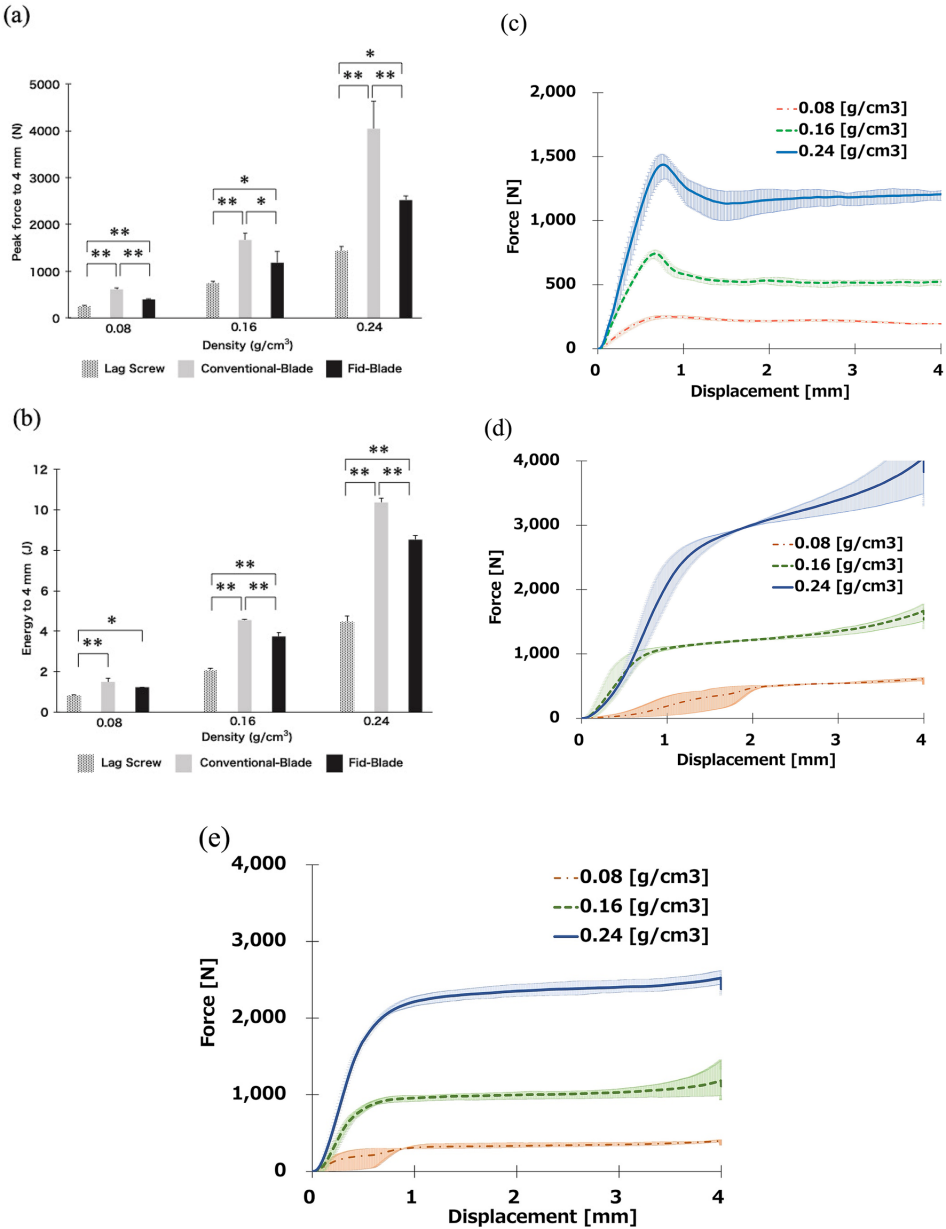
Push-in test. (**a**) Mean peak force to achieve a 4-mm displacement. (**b**) Mean energy force to achieve a 4-mm displacement. *P < 0.05. **P < 0.01. (**c**–**e**) Mean force–displacement curves. (**c**) Lag screw. (**d**) Conventional-Blade. (**e**) Fid-Blade.

Figure 4a,b show the highest penetration resistance in the push-in test. The higher the density of the polyurethane block, the higher the peak force and the energy required to overcome this resistance. The peak force to achieve a 4-mm displacement was significantly greater for Conventional-Blade (612.48 ± 26.25 N) and Fid-Blade (397.32 ± 16.93 N) than that for Lag Screw (252.19 ± 12.14 N) at a density of 0.08 g/cm^3^ (both *P* < 0.01). Moreover, the peak force to achieve a 4-mm displacement was significantly greater for Conventional-Blade than that for Fid-Blade (*P* < 0.01). The energy to achieve a 4-mm displacement was significantly greater for Conventional-Blade (1.49 ± 0.2 J) and Fid-Blade (1.23 ± 0.00 J) than that for Lag Screw (0.83 ± 0.04 J; *P* < 0.01, *P* < 0.05, respectively), with no significant difference between Conventional-Blade and Fid-Blade.

**Figure 5:**
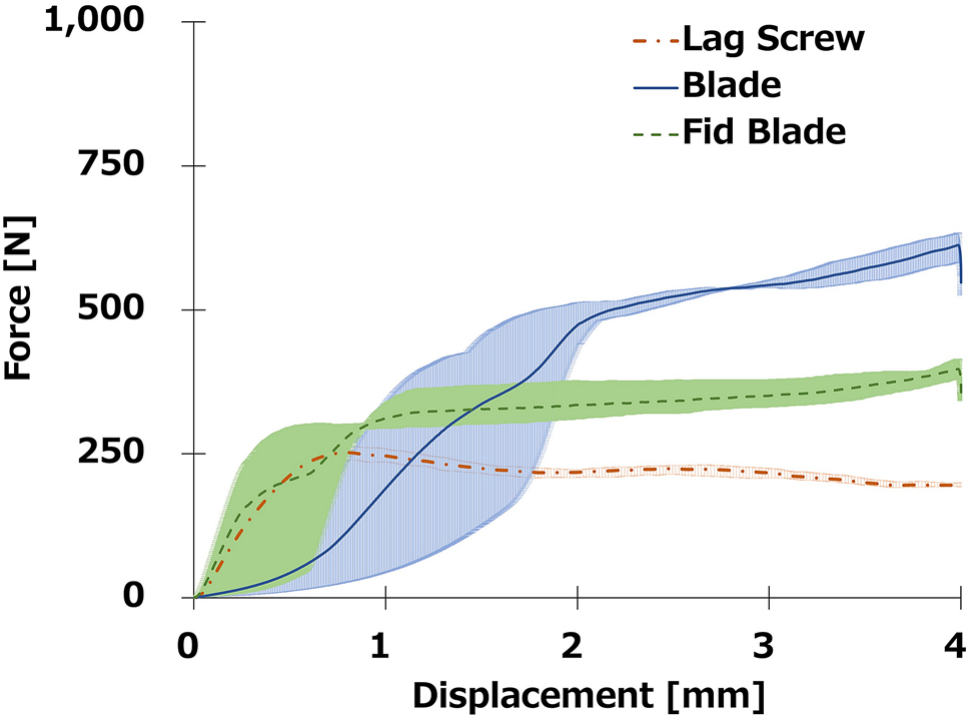
Mean force–displacement curves for the push-in test for a density of 0.08 g/cm^3^. The three components ranked in descending order, by both peak force and energy, to achieve a 4-mm displacement: Conventional-Blade > Fid-Blade > Lag Screw.

Hence, for all densities of the polyurethane block mimics, the three components ranked in descending order in terms of both peak force and energy to achieve a 4-mm displacement: Conventional-Blade > Fid-Blade > Lag Screw. The mean force–displacement curves for the push-in test are shown in Fig. 4c–e and those for the push-in test for a density of 0.08 g/cm^3^ are presented in Fig. 5. The resistance value of Conventional-Blade and Fid-Blade gradually increased with insertion, whereas that of Lag Screw decreased after reaching the highest insertion resistance value.

## Discussion

This study compared the biomechanical properties of both blade types and lag screw components of the Magnum nail in osteoporotic bone models. Conventional-Blade and Fid-Blade showed significantly higher values than Lag Screw under any of the conditions used in our study. Conventional-Blade and Fid-Blade were three to four times more resistant to rotation than Lag Screw at all densities of the polyurethane block (Fig. 3). Moreover, the energy required for the implant to displace by 4 mm was the greatest for Conventional-Blade, followed by Fid-Blade, and the least for Lag Screw under all conditions (Fig. 4). Additionally, as the insertion progressed, the fixation capability of the blade type increased, while that of the lag screw type decreased accordingly (Fig. 5).

The principles of fracture treatment include reduction and stable fixation. The importance of reduction in the treatment of trochanteric fractures of the femur, particularly unstable fractures, has been extensively reported in recent years^10–12^. Fang et al.^10^ compared 197 cases treated with DHS blade fixation and 242 cases treated with DHS fixation and reported that reduction procedures are more important than implant selection^13^. However, in cases of femoral trochanteric fracture with severe osteoporosis, bony support may be insufficient after reduction, which can cause unstable fixation of the femoral head, leading to cut-out^14^. The helical blade, which is generally used at present, is useful for stable fixation^15–17^; however, the reduction position may be rotationally displaced because the helical blade is inserted using a rotational movement. Hence, to aim for reduction and stable fixation, we believe that implant selection may be also important in the treatment of femoral trochanteric fractures with severe osteoporosis. There are three major differences between Magnum nails (Robert Reid Inc., Tokyo, Japan) and traditional implants. First, the blades of the Magnum nail have a non-spiral shape that helps avoid the rotational force to the femoral head upon its insertion. Second, the system has two types of blades: Blade (Conventional-Blade) and Fid Blade (Fid-Blade, with fewer longitudinal grooves that facilitate easier nail removal in young individuals). Finally, the surgeon can choose among the three components, including two blade-style components and Lag Screw, during surgery. Hayashi et al. reported that Magnum Fid-Blades had a stronger rotational stability than Lag Screw in mechanical test and finite element analysis^18^. However, only one type of density of the polyurethane block was compared between the Lag screw and Fid Blade not including Conventional-Blade. In this study, we used three types of bone models with different densities of the polyurethane block among three components, including Conventional-Blade.

Figure 3 shows the results of torsional test. O’Neill et al.^19^ and Gosiewski et al.^20^ performed similar torsional tests for lag screw and helical blade in 0.08 and 0.16 g/cm^3^ Sawbones bone substitute material, and our torsional test finding is similar to the results of these studies (Fig. 3). The values of peak torque and energy required to achieve 30° rotation in our study were comparable to those of previous literatures^19,20^; this suggests that our method was appropriate to compare the torsional stability of the three components (Lag screw, Conventional blade, Fid blade). In the present study, the energies required by Conventional-Blade and Fid-Blade were 1.8 and 1.48 times greater than those required by Lag Screw in a severe osteoporosis model. These results may reflect the fact that unlike the lag screw type, the other two types of blades can be inserted without prior reaming and that the bone around the implant is compacted by compressing the cancellous bone during insertion^21,22^. In addition, Conventional-Blade has a concave-shaped pentagonal structure while Fid-Blade has concave-shaped rectangular structure, and each surface which has a wider area than that of helical blade contributes to rotational stability^18^. Figures 4 and 5 show the result of push-in test. Our push-in test performed on bone models of severe osteoporosis (0.08-g/cm^3^ density) showed that Conventional-Blade had a significantly higher resistance force than the other two types (Fig. 4), and the force of the blade type increased, whereas that of the lag screw decreased with the progression of the insertion (Fig. 5). It has been reported that bone around blades is compacted by compression of cancellous bone during insertion^21,22^; therefore, as Conventional-Blade with the concave-shaped pentagonal structure has an adequate thickness for insertion without rotational movement, it could compress the bone around the blade and this contributes to its high resistance value. Goffin et al. have reported in their study that finite element models display an advantage by using a helical blade, which provides better bone purchase^21^. Our results also show that the blade type component has a higher fixation capability than lag screw type component in severe osteoporotic bone model.

**Figure 6:**
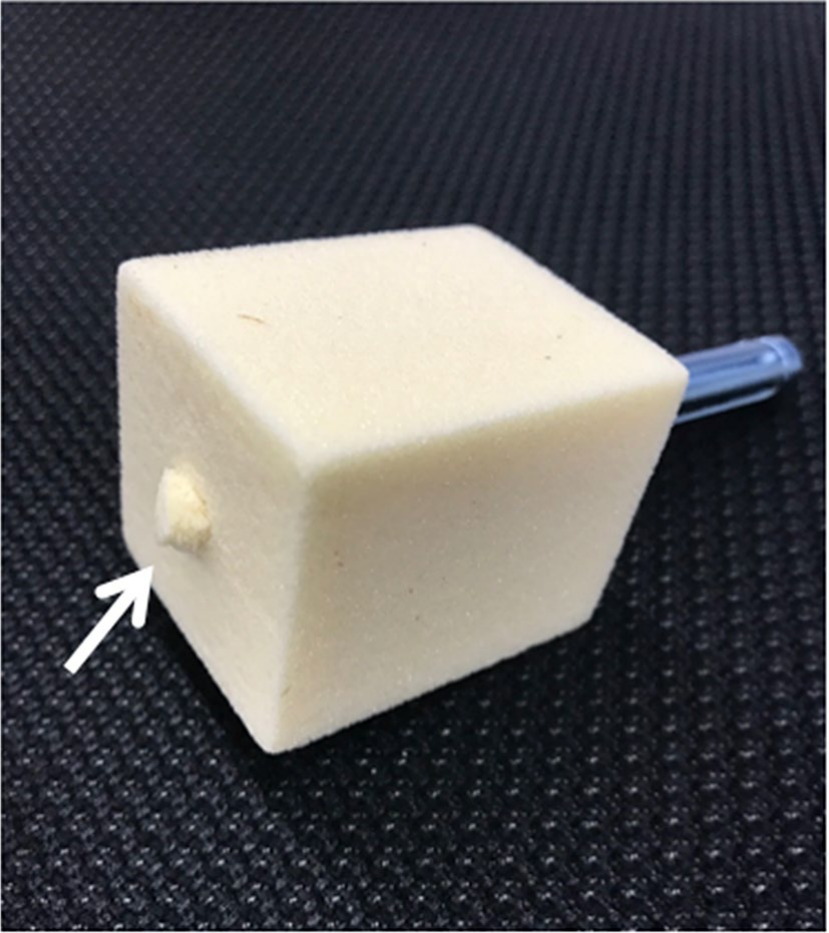
Protrusion of the nail on the surface of the polyurethane foam block following the push-in test (white arrow).

However, this result of push-in test recommends careful consideration of the difficulty of insertion and removal of the Conventional-Blade, particularly in patients with normal bone mineral density. Figure 6 shows the protrusion of the Conventional-Blade on the surface of the polyurethane foam block following the push-in test. According to Nikoloski et al. and Lenz et al., the blade type behaves differently to the lag screw type, and placement too close to the subchondral bone may lead to penetration through the head^23,24^; the result of our push-in test supports their finding. In addition, this protrusion may worsen owing to excessive intraoperative insertion force and postoperative weight-bearing and may lead to ‘cut through’, i.e. migration of implants into the pelvis, which is among the complications reported in patients with blade fixation. Goffin et al. also reported that for their finite element model that matched normal bone density, the superiority of the PFNA was relinquished^20^, similarly, our bone models that matched normal bone mineral density indicate that the high fixation capability of Conventional-Blade might lead to difficulty of insertion and loss or failure of fixation. Therefore, surgeons should be mindful of the risk of iatrogenic fractures, difficulty of insertion, and reduction loss with bone compaction and carefully consider using the Conventional-Blade, particularly in cases with normal bone density. If it is difficult to insert the Conventional-Blade; hence, pre-reaming is required. Further, pre-reaming reduces the fixation force of the blade; thus, we believe that Lag Screw can be safely used in such cases. Additionally, in younger patients with normal bone mineral density, the removal of Lag Screw and Conventional-Blade can be difficult owing to the structure of these devices. In such cases, surgeons can select Fid-Blade, which has large face shapes on the lateral surfaces that enhance rotational resistance and fewer longitudinal grooves^18^, facilitating easier nail removal. In this respect, the Magnum nail is useful, since the surgeon can choose among three components, with reference to the sensation of drilling in the femoral head.

The different biomechanical studies published so far do not seem to agree on whether this compaction effectively leads to better implant fixation capability^19,25,26^. The following are the clinical implications of our findings: blade type is better than the lag screw type in a severely osteoporotic bone; however, in patients with normal bone mineral density, the blade type (especially Conventional-Blade) should still be inserted cautiously. Thus, our results indicate that a suitable implant for fixation of the femoral head may vary according to the bone density of the patient. The various options available with the Magnum nail system allow the best implant selection for patients, ranging from young patients to patients with severe osteoporosis.

This study had some limitations. First, it used a bone model instead of a cadaveric bone. Although, it is desirable perform these comparisons in real bone, the number of real bone samples is limited, and the bone density of each sample may vary. Therefore, using real bone for the sample may make it difficult to achieve highly reproducible measurements. Meanwhile, using simulated bone as a sample may provide highly reproducible and uniform measurements in the osteoporosis model. In this comparison between lag screw type and blade type, simulated bone was considered useful. Second, we did not utilize bone models with densities < 0.08 g/cm^3^ in this study, and further studies are necessary to evaluate the fixation capability of the implants in more severe osteoporotic bone models. Nonetheless, 0.08 g/cm^3^ is the lowest density available amongst the variations of Sawbones’ polyurethane foam. Our study is important because it compares the fixing forces of non-spiral blades and lag screws using the lowest density polyurethane blocks available; however, subsequent evaluation in clinical practice is also necessary. Third, although Magnum nail has been approved by Japanese Pharmaceuticals and Medical Devices Agency (approval number: 22400BZX00436000) and is already on the market in Japan, the stiffness and strength of these implants were not examined in this study. Fourth, only a static rather than a dynamic test under conditions of an added rotational moment and axial load was performed; we conducted torsional test to examine torsional resistance—a predictor of the most common failure modes including cut-out^27^ and push-in test to examine insertion and load resistance. However, the fracture site is loaded in a multiplanar, dynamic manner during walking^28^. To clarify the cut-out risk, analysis of the effects of loadings with movement or loading point variation such as hip implant performance simulator^28^ and the interior of the hole after the insertion is necessary. This should be addressed to understand the characteristics of the implant; hence, further studies are necessitated.

## Conclusion

The findings suggest that the fixation capability of the blade-type Magnum nail component is better than that of the lag screw type in a severe osteoporotic bone model. Thus, the blade-type Magnum nail component may be useful for treating femoral trochanteric fractures in patients with severe osteoporosis.
